# SARS-CoV-2 entry into and evolution within a skilled nursing facility

**DOI:** 10.1038/s41598-023-38544-5

**Published:** 2023-07-19

**Authors:** Nicole R. Sexton, Parker J. Cline, Emily N. Gallichotte, Emily Fitzmeyer, Michael C. Young, Ashley J. Janich, Kristy L. Pabilonia, Nicole Ehrhart, Gregory D. Ebel

**Affiliations:** 1 Department of Microbiology, Immunology and Pathology, College of Veterinary Medicine and Biomedical Sciences, Colorado State University, Fort Collins, CO 80523 USA; 2 Nebraska Center for Virology, School of Biological Sciences, University of Nebraska-Lincoln, Lincoln, NE 68504 USA; 3 Columbine Health Systems Center for Healthy Aging and Department of Clinical Sciences, Colorado State University, Fort Collins, CO 80523 USA

**Keywords:** SARS-CoV-2, Evolution, Viral epidemiology

## Abstract

SARS-CoV-2 belongs to the family Coronaviridae which includes multiple human pathogens that have an outsized impact on aging populations. As a novel human pathogen, SARS-CoV-2 is undergoing continuous adaptation to this new host species and there is evidence of this throughout the scientific and public literature. However, most investigations of SARS-CoV-2 evolution have focused on large-scale collections of data across diverse populations and/or living environments. Here we investigate SARS-CoV-2 evolution in epidemiologically linked individuals within a single outbreak at a skilled nursing facility beginning with initial introduction of the pathogen. The data demonstrate that SARS-CoV-2 was introduced to the facility multiple times without establishing an interfacility transmission chain, followed by a single introduction that infected many individuals within a week. This large-scale introduction by a single genotype then persisted in the facility. SARS-CoV-2 sequences were investigated at both the consensus and intra-host variation levels. Understanding the variability in SARS-CoV-2 during transmission chains will assist in understanding the spread of this disease and can ultimately inform best practices for mitigation strategies.

## Introduction

The SARS-CoV-2 pandemic began traversing the world in 2019 and rapidly established local transmission cycles on every continent. While SARS-CoV-2 can infect humans of all ages, risk of severe infection and death increases non-linearly with age^1,2^. In the United States as of 09-19-2022, 1,048,301 deaths have been attributed to infection with SARS-CoV-2. Importantly, 93.3% of these deaths have been in adults 50 years of age and over, who make up 35.7% of the US population. Conversely, only 6.7% of deaths from Covid-19 disease have been in individuals under 50, at 64.5% of the US population, (“https://covid.cdc.gov/covid-data-tracker/,” n.d.). Overall, risk of severe morbidity and mortality from SARS-CoV-2 infection increases exponentially as humans age^3^. As a result of combining the age stratified risk and asymptomatic/pre-symptomatic spread of SARS-CoV-2^4–7^ with advanced age, grouped housing, required living assistance, and routine transfer to and from hospitals of individuals living in skilled nursing and rehabilitation facilities, these congregate care settings have borne some of the heaviest burdens of the pandemic in regard to mortality as well as from restrictive policy decisions^1,8–10^. This is consistent with what has been established for the other endemic coronaviruses which also result in hospitalizations^11–13^ and outbreaks in skilled nursing facilities, albeit at reduced loads^14^. Therefore, understanding how SARS-CoV-2 enters, and spreads within, skilled nursing facilities is of extreme importance both for SARS-CoV-2 directly as well as for understanding if other coronavirus outbreaks can be mitigated in these settings.

SARS-CoV-2 is a nidovirus, which include the largest known positive-sense RNA viruses with genomes ranging from ~ 20 to 41 kilobases^15–19^. To maintain exceptionally large RNA genomes nidoviruses encode for a proofreading exoribonuclease, leading to increased replicative fidelity^20–24^. As a result, mutation accumulation in SARS-CoV-2 is slow^25^. SARS-CoV-2 genomes from connected transmission events often present with identical genomes. Mutations that are observed across SARS-CoV-2 virus samples display skewed mutational profiles that are driven by genome editing through cellular processes. In particular, APOBEC deamination of cytosine results in C-to-U mutations, ADAR deamination of adenine drives an increase in A-to-G mutations, and ROS oxidation of guanine leads to G-to-U mutations (along with reciprocal G-to-A, U-to-C, and C-to-A mutations respectively)^26^. Large scale investigations of sequenced genomes, including minority variants demonstrate little intra-host genetic diversity^27–29^. Intra-host diversity has been found to increase with increasing duration of infection and is inversely related to viral titers^30^. Due to observed limited genetic diversity particularly at the consensus level, but also at the level of minority variants, tracking transmission chains is not straightforward and will require a deeper understanding of genetic changes though direct transmission events.

Here staff and residents of a skilled nursing facility were prospectively tested for SARS-CoV-2 by RT-qPCR. The facility was enrolled for weekly surveillance testing regardless of symptoms, beginning before any cases had been identified. Full SARS-CoV-2 genomes were sequenced from all positive samples during the initial outbreak in this facility, including samples from subjects positive over multiple testing dates. Minority variants were also assessed. The outbreak in this Colorado facility was captured from the first known infected staff member and continued throughout a large-scale outbreak within the facility that included both residents and additional staff members. Sequencing data demonstrates that the first several individuals identified as positive for SARS-CoV-2 did not initiate the interfacility outbreak but instead resulted in no or very few transmission events. The linked interfacility outbreak was sudden, with many individuals identified simultaneously and no clear indication of a patient zero for the connected genotypes. Additionally, once introduced the outbreak persisted over several weeks with nearly all identified positive samples linked to the initial cases. Minority variants were linked across individuals and were most closely associated with time of sampling, which was not observed across non-epidemically linked samples. Most minority variants disappeared rapidly from the population but one set rapidly fixed and persisted in a subset of subjects. Together, these data support a model where transmission is minimal on an individual level but where bursts of transmissions are still occurring. Investigating the mechanisms that lead to these multi-subject transmission events will be essential to preventing future devastation in skilled nursing and rehabilitation facilities.

## Methods

### Human participants

Testing at Facility G began on June 8th, 2020. This study was reviewed and approved by the Colorado State University IRB under protocol number 20-10057H, with all methods performed in accordance with the provided guidelines and regulations. No human reads were assessed, only virus present in nasal swabs from surveillance. Like previous studies, informed consent was obtained, and participants were promptly informed of all test results. Healthcare workers represented all job classifications, including those in direct patient care roles (doctors, nurses, physical therapists, etc.) and nondirect patient care roles (custodial, administrative, etc.)^4,5^. Residents of the skilled nursing facility included both long-term and transient patients. During the study period facility G had 87 staff members and 63 residents.

### SARS-CoV-2 vRNA surveillance testing

Nasal swabs were collected, processed, and tested for viral RNA as described previously^4,5^. Briefly, swabs were collected by trained personnel and placed in tubes containing viral transport medium. RNA was extracted and quantitative reverse transcriptase PCR (qRT-PCR) was performed using the CDC 2019-nCoV primers and probes^31^ or the Thermo Fisher Scientific TaqPath COVID-19 combo kit, under the U.S. Food and Drug Administration (FDA) Emergency Use Authorization (EUA) to identify positive individuals. An aliquot of samples that were identified as positive during the above routine testing were received for re-RNA extraction and subsequent full genome sequencing.

### SARS-CoV-2 whole-genome sequencing

Sequencing was performed as previously described^4,5^. Briefly, RNA from positive individuals was re-extracted, cDNA was generated in duplicate using SuperScript IV, PCR amplification was performed with V3 ARTIC tiled primers and Q5 High-Fidelity polymerase, with a decreased annealing temperature of 63C. PCR products were purified, and libraries were prepared using KAPA HyperPrep kit and unique dual index primers. Libraries were sequenced on the Illumina MiSeq platform on the V2 2 × 250 paired-end kit with all samples sequenced in duplicate.

### Bioinformatic processing of SARS-CoV-2 sequences for consensus and minority variant calling

FASTQ files from the MiSeq were aligned using BWA-mem^32^. Consensus sequences for alignments were generated in Geneious using the Highest Quality (60% Threshold, Assign Quality Total, if no coverage call ref, Trim to reference sequence and Call Sanger Heterozygotes 60%, for samples with coverage greater than 95%. Alignments were generated in Geneious using Geneious Map to Reference Mapper with High Sensitivity, iterate up to 5 times, do not trim. Areas with minor gaps were masked before processing for phylogenetic trees. This was followed by generation of a PHYML tree with Tamura-Nei substitution model and 100X bootstrapping. The resulting alignment was condensed to one line per unique genome. Treetime maximum likelihood phylodynamic analysis software was used to generate phylogenetic trees and for analyses of consensus mutational frequencies using pre-set settings github.com/neherlab/treetime_web, treetime.biozentrum.unibas.ch^33^. The iVar variant caller, github.com/andersen-lab/ivar, was used to trim primers from BWA aligned sequences using default quality threshold of 20, sliding window 4 and to generate consensus sequences (min depth 10) to identify minority variants with a cutoff of 3%^34^. Minority variant mutational compositions analyzed in Microsoft Excel and Graphpad Prism.

### Ethical approval

This study was reviewed and approved by the Colorado State University IRB under protocol number 20-10057H. Participants were promptly informed of all test results and only data related to viruses were investigated.

## Results

### Prospective sampling of staff and residents at a skilled nursing facility demonstrate multiple introductions followed by a sustained outbreak

Multiple skilled nursing and rehabilitation centers in Colorado, were simultaneously enrolled in SARS-CoV-2 screening in an attempt to limit transmission in these vulnerable populations. While all residents and dedicated staff were tested weekly, many individuals are involved in the maintenance of a facility, including transient personnel such as delivery drivers. Additionally, residents leave and are added to facilities and staff members routinely work at multiple facilities, contributing to interfacility transmission events. Skilled nursing and rehabilitation centers are dynamic. Further, weekly screening fails to identify individuals with shorter infectious cycles. Here we focus on one facility, Facility G, in the cohort where an outbreak was captured robustly, and compare it to other facilities, Facilities I, J and H, found in the same metro area.

**Figure 1:**
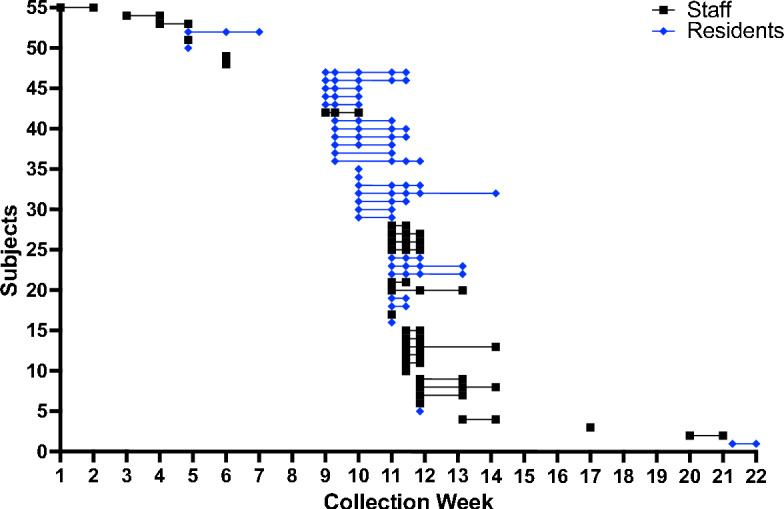
Timeline of positive samples identified in Facility G over time. Staff and residents were swabbed and tested for presence of SARS-CoV-2 by qRT-PCR at least weekly. Positive samples are graphed by individual across time.

Beginning June 8th, 2020, staff and residents within a single facility were screened weekly by qRT-PCR to identify asymptomatic and pre-symptomatic infected individuals. On June 16th, 2020, the first positive healthcare worker was identified. During these studies 22-week duration 44.4% of the residents and 31% of the staff members in Facility G were identified as positive for SARS-CoV-2, nearly all of these within a 6 week span of time. Following the first healthcare worker, positive individuals were identified through week seven but the numbers infected were low per week. At the time of sampling on week eight, no healthcare workers or residents were identified as positive, but the reprieve was short lived and week nine began a burst of infection that would last for the next 5 weeks infecting at least 44 additional individuals (Fig. 1). Given the lack of identified new positive individuals on week eight, it was unclear whether this represented one sustained outbreak that began on June 16th, or multiple successive introductions to the facility, therefore we sought to sequences all positive samples collected from this facility.

**Figure 2:**
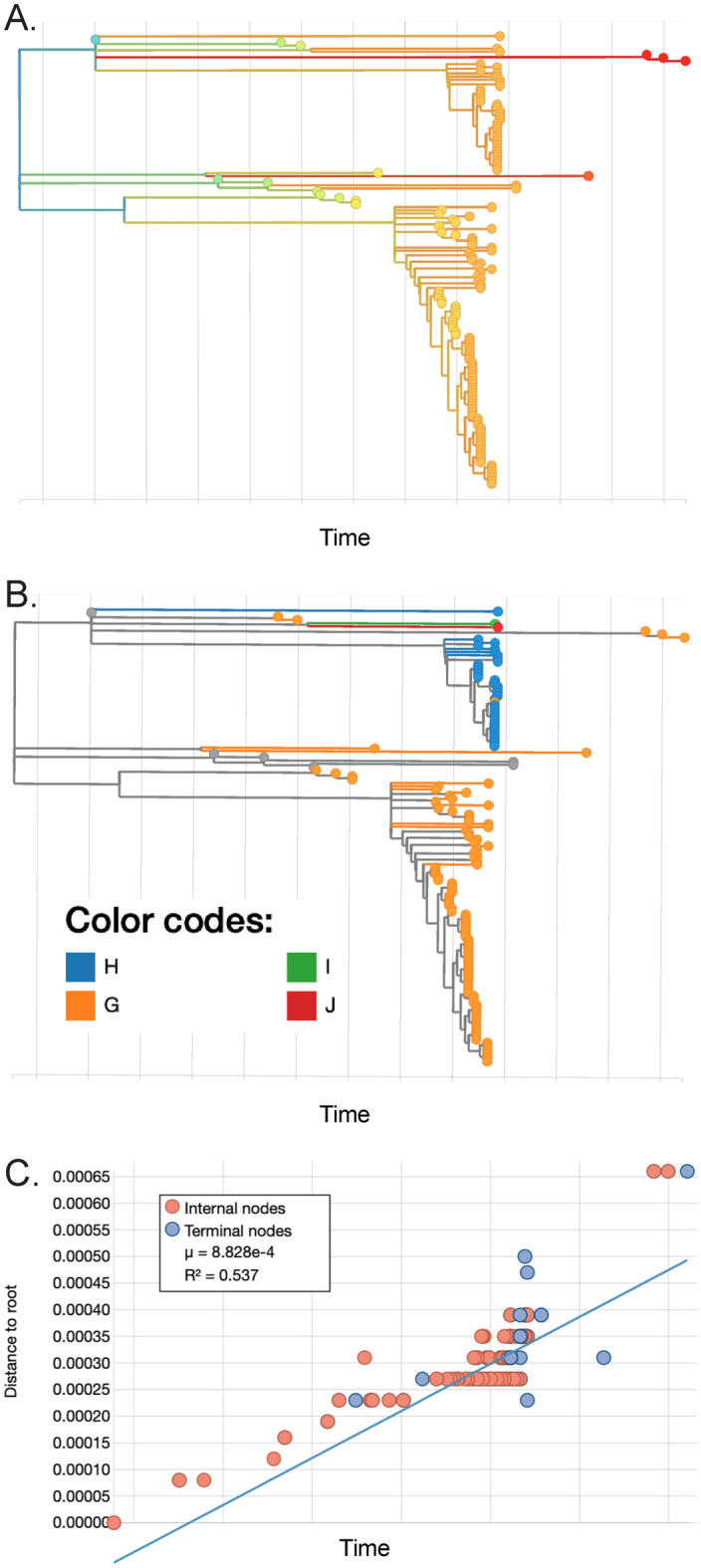
Multiple separate introductions of SARS-CoV-2 to Facility G, with one intrafacility outbreak. PHYML Timetrees colored by (**A**) time with blue for earliest sample emergence and transitioning to red for the latest, or (**B**) by facility G (orange), H (blue), I (green), J (red) or reference sequences (gray). (**C**) Distance to root graph over time for samples present in the phylogenetic analysis.

To determine if incident infections were the result of sustained transmission where sampling was inadequate to catch intermediate transmission events or if the observed pattern of infections was representative of multiple dead-end introductions followed by one or multiple large introductions, positive samples were prepared for full-genome high-throughput sequencing. Additional facilities, Facility H, I and J, were included to provide out groups occurring at the same sampling time, and in the same geographic locations in Colorado. Phylogenetic analysis of Facility G samples demonstrate that the earliest samples are not closely genetically related to the later grouped samples from this outbreak (Fig. 2A,B). In fact, the data support at least five separate introductions into the facility from two distinct viral clades. The first, third, and fifth introductions, along with some samples collected from facility G after this initial outbreak subsided, cluster more closely with the outgroup facilities H, I, and J (Fig. 2A,B). Distance to root data support evolution occurring at a consistent rate over time, supporting separation between phylogenetic clades (Fig. 2C). In this data set, most introduction into the facility didn’t result in any further spread or resulted in very few additional individuals infected. The large outbreak appears to have resulted from the introduction of a single genetic lineage at a single time point, suggestive of a super-spreading individual or event.

### A secondary group within the facility G outbreak is linked by a co-transmitted double mutation, with one mutation in nsp3 and the other in nsp13

**Figure 3:**
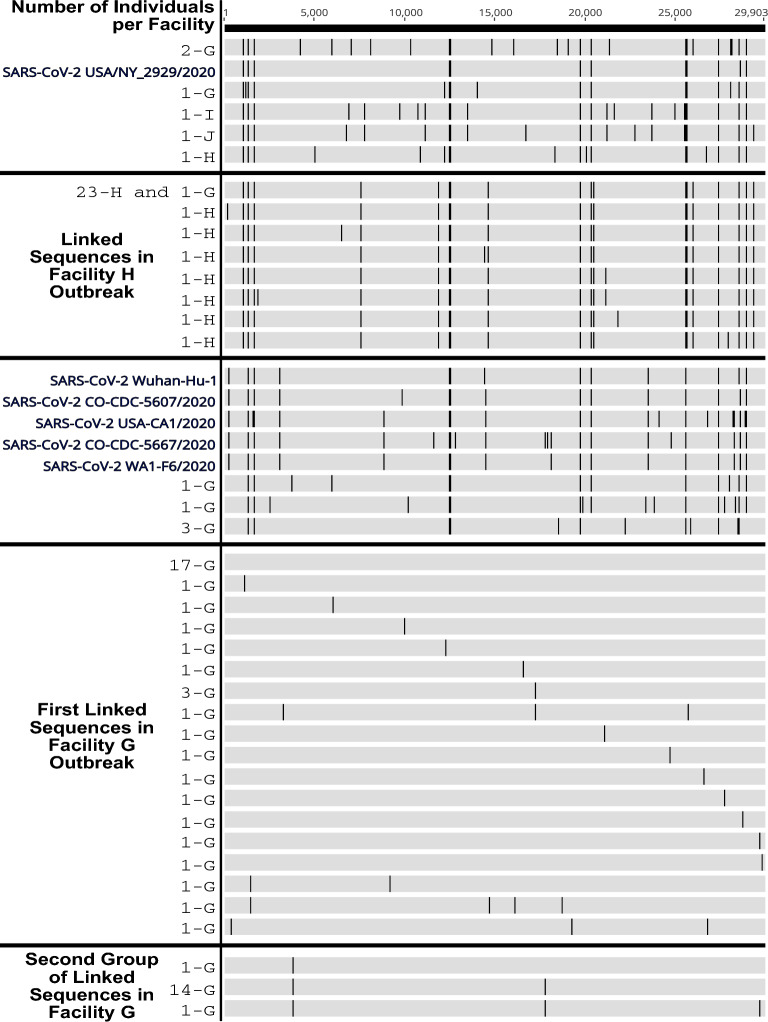
Facility G infections belong to two sequence groups with few mutations present between samples. SARS-CoV-2 sequences mapped to reference genome. Mutations that differ from the consensus sequence of the core Facility G outbreak shown as a dark line. Reference sequences shown in blue. The number of unique individuals carrying shown sequences and the facility those individuals worked or lived in are shown (#—Facility Letter, i..e. 17-G is 17 individuals in facility G with identical sequences).

Consensus sequences from all facility G, H, I and J samples, were aligned to the Wuhan-Hu-1 (NC_045512.2) reference sequence. As demonstrated by the phylogenetic tree (Fig. 2), alignment of the facility G sequences can be divided into two main groups. The first group includes the early and late facility G introductions that cluster closely with either a reference sequences from New York (USA/NY_2929/2020), or reference sequences from California (USA-CA1/2020, MT304486.1), Colorado (CO-CDC-5607/2020, and CO-CDC-5667/2020), and Washington (WA1/2020, MT246667.1), as well as infections at unrelated skilled-nursing facilities. The second group identified consists exclusively of linked sequences within Facility G suggesting an intrafacility outbreak (Fig. 3). Seventeen individuals were infected with identical sequences, with other sequences only one to four mutations away from the consensus. The sequences 3 and 4 mutations away from the consensus in this group belonged to individuals’ late time point samples and are not representative of separate introductions. The alignment also highlights a subgroup of 15 individuals within the interfacility outbreak that include genomes with two co-transmitting synonymous mutations (C3796U and C17766U) distant from each other in the genome. Only one sample had a consensus level mutation called in one of these two sites (C3796U) without the other; however, upon closer inspection of the data, this sample has both mutations, but the second mutation falls below consensus at ~ 30.2%, suggesting that these two mutations were consistently transmitted collectively. Expectedly, individuals with multiple positive samples maintain consistent consensus genomes over time. Resident (R) R508 is the exception in this data set, with multiple mutations fixed by the fourth sample on 9/1/2020, where the first positive sample was collected on August 19th. This is consistent with observations in other studies demonstrating late time points accumulate more mutations^30^ and was similarly observed in our previous data sets^4,5^. Only one other mutation links a subset of subjects from within the outbreak consensus group with mutation G17218A observed in two residents and two staff members. Though, one staff member carried two additional consensus mutations. Overall, these data suggest few transmission events occurred within the facility with multiple introductions. However, intrafacility outbreaks still occur, likely the result of super-spreading events or super-spreading individuals within the facility.

### Consensus mutation frequencies trend towards conformation to broad data sets

**Figure 4:**
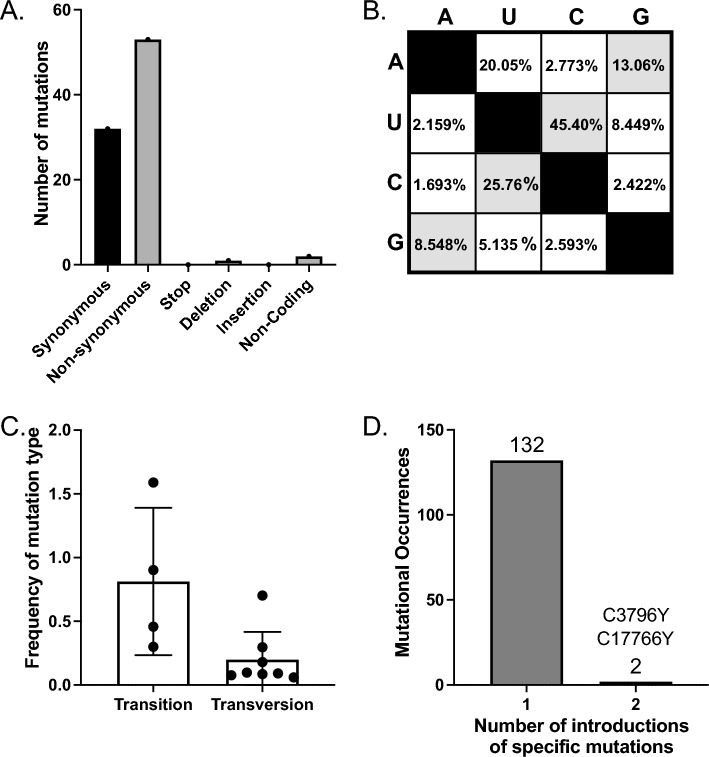
Analysis of consensus mutations present across all samples with the phylogenetic analysis. (**A**) Types of consensus variants observed: synonymous, nonsynonymous, stop codon, deletions, insertion, or non-coding. (**B**) Mutation frequencies observed from vertical nucleotide to horizontal nucleotide. (**C**) Transitions vs. transversion fixed in the population sequenced. Each dot represents a specific transition (e.g., X → Y) or transversion (e.g., X → Y). Error bars SD. (**D**) Number of times specific mutations were likely introduced in the data set determined by TimeTree analysis.

Mutations that fixed in the SARS-CoV-2 genome of positive individuals in the skilled nursing facilities surveyed were nearly exclusively synonymous or nonsynonymous mutations with no stop codons or insertions detected above 50% percent of a virus population (Fig. 4A). Two non-coding mutations were identified at consensus levels and one deletion. The consensus level deletion was identified in R524 on 09/01/20 removing an adenine from position 16,148 in the NiRAN domain of nsp12 and made up 52.3% of the population. This is a nonsense mutation that should result in nsp13–16 not being generated, suggesting that more than half of the viral population sequenced from this individual’s final timepoint consisted of defective viral genomes. While surprising to have one nonsense mutation found at such a high percentage of the viral population, this may be a function of declining SARS-CoV-2 populations at the end of infection and relate to increased mutations routinely identified in final timepoints.

SARS-CoV-2 mutational frequencies are not fully consistent with standard transition vs. transversion biochemistry but are influenced by host cell deamination by APOBEC and/or ADAR, and oxidation by ROS^26^. Within our data set at consensus levels, we see skewed mutational frequencies with particularly high numbers of U–C (ADAR), C–U (APOBEC), and A–G (ADAR) transitions, and A–U transversions (Fig. 4B,C). This is like what is seen across larger outbreaks but doesn’t match perfectly, which is to be expected with a small sample size. Similarly, while most mutations appear to have been introduced into the population only once across the samples investigated here, the linked mutations C3796T and C17766T were fixed twice (Fig. 4D). We next assessed minority variants present across Facility G samples.

### The composition of minority variants across facility G is consistent with global SARS-CoV-2 evolution

**Figure 5:**
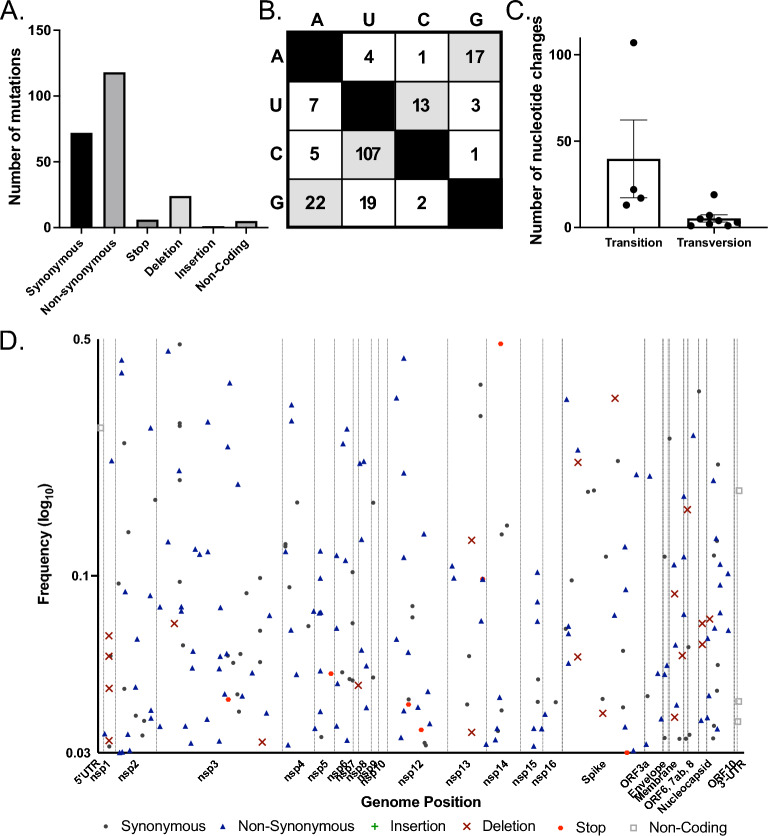
Analysis of minority variants present across facility G samples. (**A**) Types of minority variants observed: synonymous, nonsynonymous, stop codon, deletions, insertion, or non-coding. (**B**) Specific minority variant mutations from vertical nucleotide to horizontal nucleotide. (**C**) Observed transitions vs. transversions across samples. Each dot represents a specific transition (e.g., X → Y) or transversion (e.g., X → Y). Error bars SD. (**D**) Variants by frequency across the genome. Log scale x-axis starts at 0.03 (3%).

Assessing minority variants can identify mutational introductions before removal by selection pressures, such as stop codon introductions that are not be found at consensus levels. PrimalSeq using ARTIC Network primers was performed in duplicate across all facility G samples, sequenced on the Illumina MiSeq platform, and analyzed using the iVar bioinformatic pathway^34^. Overall, individual samples had minimal minority variants presents, consistent with the low mutation rates of Coronaviruses because of proofreading by nsp14-ExoN^23,24^. Minority variants across facility G were combined to assess the types of mutations being generated and whether there were locations across the genome where mutations were identified more frequently. In contrast to mutations observed at the consensus level, minority variants included an insertion, as well as multiple deletions, and stop codons, though less frequently than synonymous and nonsynonymous mutations (Fig. 5A). The iVar bioinformatic process is most accurate for mutations present in the population above 3%, so we used this cut off. We additionally, discarded any insertions or deletions of a single U, as these are nearly exclusively found in poly-U sites and are known sites of sequencing error. We also discarded all minority variants that didn’t pass the iVar platforms statistical cut off for acceptance for both replicates. Across the acquired sequences there were an average of 4 minority variants per sample (s.d ± 6.7).

Mutation types across minority variants retained in this analysis recapitulate the mutation types observed across global SARS-CoV-2 viruses sequenced and support the roles of APOBEC, ADAR, and ROS in the driving of specific types of mutations. The most frequent mutations observed are C–U transitions at 53.2% (APOBEC), additionally G to A mutations account for 10.6%, A–G and U–C mutations (ADAR) represent 8.5% and 6.5% of mutations respectively, and finally G–U and C–A mutations (ROS) represent 9.5% and 2.5% of mutations (Fig. 5B,C). The rest of the mutation types combined make up only 16.94% (Fig. 5B), and these numbers are again similar to previous reports across global consensus changes^26^. Overall, transitions contribute far more to the diversity of SARS-CoV-2 than transversions (Fig. 5C). All consensus mutations were introduce to the facility only once, except the pair of mutation C3796U/17766U which were introduced at consensus levels twice (Fig. 5D).

### The synonymous mutations C3796U and C17766U were present in multiple individuals at matched timepoints

**Figure 6:**
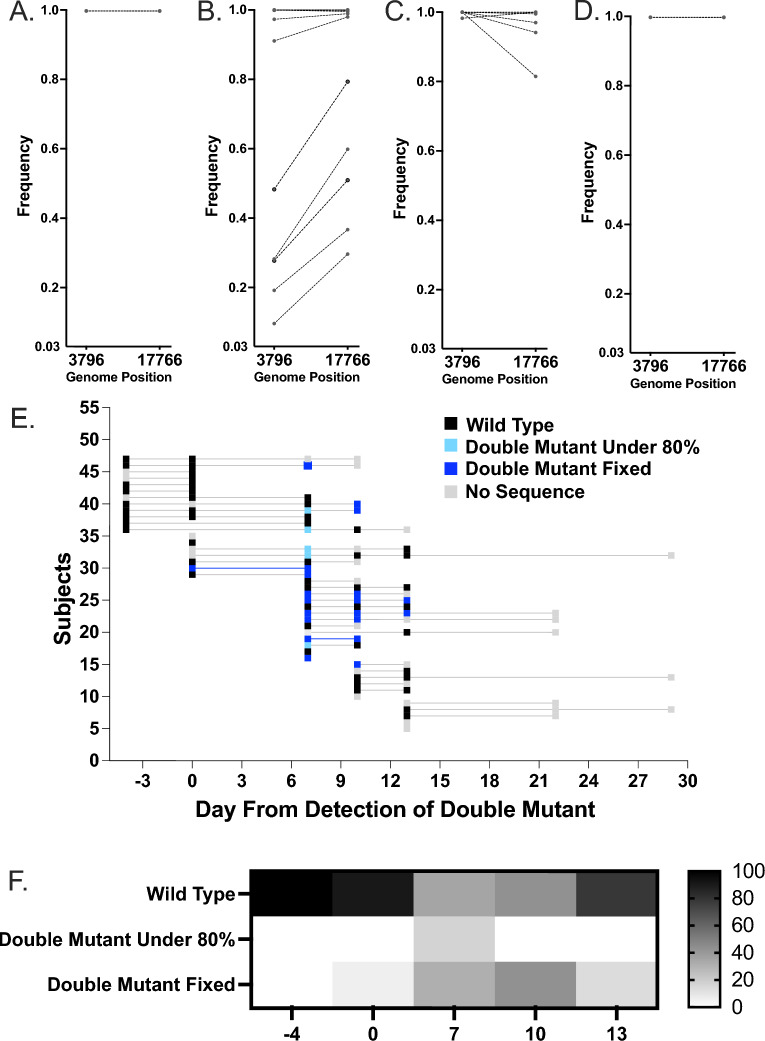
Within facility G two mutations are found together across individuals: C3796U and C17766U. Frequency of mutations in individual’s virus population by weeks post first SARS-CoV-2 introduction (**A**) 0, (**B**) 7, (**C**) 10, and (**D**) 13. Lines link minority variant found within the same sample. Frequency starts at 0.03 (3%). (**E**) Presence of WT vs double mutant in sequences by subject over time. (**F**) Percent of WT vs. double mutant present in sequences.

A subset of individuals within facility G were found to have a pair of synonymous mutations present at consensus levels, C3796U and C17766U (Figs. 3 and 6A–D). We next analyzed the frequency, date, and subjects for these mutations at both consensus and minority levels. The first time C3796U and C17766U mutations were identified in the virus population of a nasal swab was on week ten (8/19/2020) in a single resident at fixation (Day 0) (Fig. 6A,E). In week eleven, day 7 post-identification, (08/24-25), C3796U and C17766U were identified in 14 out of 26 subjects (Fig. 6E). These mutations were found exclusively as a pair within virus populations and were identified at frequencies varying from 9.6 to 100% (Fig. 6A–D). Interestingly, two subjects were identified with this set of mutations present at fixation and four as a portion of the viral population on 8/25/20 who did not have detectable levels of either of these mutations in samples sequenced at previous time-points, suggesting either that these individuals either had these mutations present in virus populations at very low levels, separately selected for this same group of two synonymous mutations, or that they were superinfected by an individual harboring this set of mutations. Most individuals with theses mutation present below fixation lost them before sampling on 8/28/20, but one fixed in the virus population (Fig. 6E). Overall, these data support intrafacility spread and potential connections between specific individuals. However, the data also demonstrate the complexities of SARS-CoV-2 evolution across a human population and demonstrate a swelling and contraction of mutational accumulations as the virus spreads from individual to individual, both for minority and consensus level variants.

### Variants found in multiple samples are temporally linked within the outbreak

C3796U and C17766U were only found below fixation at a single sampling time on week 11 post SARS-CoV-2 introduction to facility G, 7 days post-introduction (Fig. 6F). We next sought to determine if minority variants found in multiple samples were similarly linked by time or subject. Ten mutations were found in multiple samples from across facility G at over 3% of individual’s virus population. We compared this to variants identified in subjects from a different facility (H) that presented with a less sequential outbreak and did not observe repeated minority variants in that population. In facility G, one subject retained three minority variants over sampling time and a different individual retained one. Thus, most minority variants are transient within individuals. Seven of the minority variants identified in multiple samples were found only in samples collected on the same date from different individuals. These were shared by between 2 and 5 individuals on either 8/25/20 or 8/28/20. In total ten subjects shared minority variants with other members of the Facility G community. Overall, this suggests that multiple genomes are likely routinely being passed during transmission events, though minority variants are subsequently lost.

## Discussion

Patients within skilled-nursing facilities have been severely affected by the SARS-CoV-2 pandemic^8,9,35^. As a result of the disproportionate effect of infection by SARS-CoV-2 in this population, extensive biosecurity standard operating protocols were altered with the intention of preventing introduction and spread among staff and residents^35^. However, despite all mitigation efforts implemented, SARS-CoV-2 continued to result in high fatality counts across facilities^1,36,37^. Our data suggest that most unique introductions of SARS-CoV-2 into skilled nursing facilities with mitigation measures such as those at Facility G did not result in outbreaks within the facilities. Which, if any, of the measures prevented spread from these introductions remain to be determined, but overall, these data demonstrate that many viral introductions were dead ends within a facility. It is also possible that none of the implemented measures were successful, and the observed pattern is only the result of known patterns of superspreading and overdispersion in transmission^38,39^. Unfortunately, our data also demonstrate that despite many introductions of the virus being limited in spread, events were still occurring that resulted in rapid widespread transmission within facilities (Figs. 1 and 2). Also, once SARS-CoV-2 is present within a facility, stopping further transmission can be difficult even with testing occurring every two to three days and recommended mitigation strategies in place. These data support a need to reevaluate the mitigation strategies used for skilled nursing facilities during future pandemics. Further emphasizing this conclusion, these data reflect SARS-CoV-2 from before the emergence of the more transmissible delta and omicron variants^40^.

Coronaviruses encode for a proofreading exonuclease^23,24,41^, as a result they incorporate fewer mutations per site per round of replication compared with most positive-sense RNA viruses^24,42^. However, replicative fidelity doesn’t always translate to observed mutations in viruses across host populations as many other factors influence the mutations that are observed, most notably selection^43–45^. Since SARS-CoV-2 was still new to the human population when these samples were collected it was yet to be determined whether selective pressures would increase the observed frequency of mutations between subjects. Additionally, studies of the virus in large data sets may enhance the mutations observed over a given time frame in a population as the vast number of individuals being infected provides more opportunities for mutations to accumulate than is observed in viruses that must traverse more linear paths through a population. Our data demonstrate that at both the consensus and the minority variant levels, SARS-CoV-2 incorporates very few mutations on average at it moves through a connected population. This makes determining transmission pathways through a population difficult as many individuals carry identical viruses by consensus sequences (Fig. 2). Minority variants may be able to support additional connections between individuals in an outbreak as groups of variants were identified in subjects linked by both facility and time of infection. However, even here variants fluctuate both within individuals and overtime, making transmission chains impossible to confirm. Still, the combination of consensus and minority variant tracking combined with inter-facility behavioral data in the future could be an optimized way of identifying where mitigation strategies have fallen short.

Viruses are typically described as evolving within single individuals with transmission of only a consensus. However, these data support transmission of virus population sizes large enough to maintain minority variants across multiple subjects during transmission events. Estimates of SARS-CoV-2 bottleneck sizes during transmission have varied but tend to suggest multiple virus particles initiating infections^28,30,46^, these data don’t allow for a quantitative measure of bottleneck size but support transmission of multiple particles due to the presence of shared minority variants across subjects. Further, the co-transmission of C3796U and C17766U suggest that viral evolution may occur collectively across host populations rather than exclusively within individual hosts. This across population evolution would likely benefit SARS-CoV-2 by allowing beneficial mutations that don’t fix rapidly enough during infection of a single host further opportunities to fix in the viral population. Additionally, this replication strategy would prevent dead ends to beneficial mutations because of unique individual host pro- or anti-viral environments. Further research into virus evolution as a function of a host population rather than during single host infections will be an exciting area of future study.

SARS-CoV-2 has proven to be a highly successful human pathogen. Its outsized effect in aging populations requires an understanding of spread that will allow for successful mitigation strategies to be implemented in the unique circumstances of skilled-nursing facilities, which have experienced difficulties overcoming challenges in protecting these populations from a highly transmissible virus. Additionally, many strategies implemented caused alternate harms for residents that are not sustainable^8,9^. Therefore, it is of the utmost importance that transmission dynamics are understood and mitigation strategies are tested to determine the best mitigation strategies while minimizing harms from strategies that aren’t successful. These data are a small start to that essential goal.

## Conclusions

SARS-CoV-2 has had a disproportionally severe impact on aging individuals living in congregate care facilities. Here we investigate the evolution of SARS-CoV-2 within these care settings to inform the pathways taken by this pathogen from a genetic perspective. We demonstrate that spread occurs in bursts despite most introductions to a facility resulting in no or limited spread. Additionally, we demonstrate that evolution likely occurs at both the individual and population levels as minority variants are spread alongside consensus genomes. Understanding how these bursts of infections continue to occur within facilities could improve future mitigation strategies.

## Data Availability

The sequences generated for this manuscript are freely and publicly available on NCBI GenBank (submission number SUB12211924, Accession Numbers: OP724412-OP724550).
